# Breaking the cycle with trauma-focused mentalization-based treatment: theory and practice of a trauma-focused group intervention

**DOI:** 10.3389/fpsyg.2024.1426092

**Published:** 2024-09-13

**Authors:** Maaike L. Smits, Jasmijn de Vos, Eva Rüfenacht, Liesbet Nijssens, Lisa Shaverin, Tobias Nolte, Patrick Luyten, Peter Fonagy, Anthony Bateman

**Affiliations:** ^1^De Viersprong, Viersprong Institute for Studies on Personality Disorders, Halsteren, Netherlands; ^2^Department of Psychiatry, Section of Medical Psychology and Psychotherapy, Erasmus MC, Rotterdam, Netherlands; ^3^Department NPI Centre for Personality Disorders, Arkin Mental Health, Amsterdam, Netherlands; ^4^Division of Psychiatric Specialties, Department of Psychiatry, Geneva University Hospitals, Geneva, Switzerland; ^5^Research Department of Clinical, Educational and Health Psychology, University College London, London, United Kingdom; ^6^Faculty of Psychology and Educational Sciences, KU Leuven, Leuven, Belgium; ^7^Tavistock Trauma Service, Tavistock & Portman NHS Foundation Trust, London, United Kingdom; ^8^Anna Freud, London, United Kingdom

**Keywords:** trauma, mentalization, mentalizing, posttraumatic stress disorder (PTSD), complex PTSD, borderline personality disorder

## Abstract

Trauma-Focused mentalization-based treatment (MBT-TF) is an adaptation of mentalization-based treatment (MBT) specifically developed for patients suffering from attachment or complex trauma, with the possibility of co-occurring borderline personality pathology. The creation of MBT-TF was driven by previous research and observations that interventions centered on mentalizing could be significantly improved by directly addressing the impact of trauma. MBT-TF aims to mitigate symptoms that arise post-trauma, such as hyperarousal, hypervigilance, intrusions, flashbacks, avoidance behaviors, dissociative experiences, negative perceptions of self and others, and ensuing relational difficulties. Implemented as a group intervention, MBT-TF typically spans 6–12 months. From a mentalizing perspective, trauma, particularly attachment trauma, leads to a failure in processing the effects of trauma through and with others. Stress and attachment behavioral systems are disrupted, which undermines the capacity for epistemic trust, and impairs mentalizing abilities. This paper offers a concise summary of the reasoning for MBT-TF’s creation, its theoretical underpinnings, and its clinical strategy for addressing the adverse impacts of trauma. It further details the treatment phases, their main goals, and their interventions, supplemented by clinical case examples that underscore MBT-TF’s distinctive attributes and frequent clinical hurdles.

## Introduction: the rationale for MBT-TF

A significant proportion of mental health patients report having experienced adversity during childhood and later life (Horowitz et al., 2000; Lippard and Nemeroff, 2020; McKay et al., 2021). Studies have consistently highlighted a strong link between such adversity and various forms of psychopathology, noting that trauma significantly influences current functioning and treatment outcomes (Horowitz et al., 2000; Huang et al., 2020; Panagou and MacBeth, 2022; Bateman et al., 2023b). Trauma, as we define it in this context, represents not solely an ‘adverse event’ or ‘adverse experience’ *per se*, but also refers to the consequences thereof. We understand trauma as an experience in which adverse events are of an intensity that is beyond the capacity of the individual to cope with. Complex trauma specifically refers to the impact of repetitive, prolonged early negative life experiences involving neglect or abuse, typically within an attachment/caregiving context or within other interpersonal relationships with an uneven power dynamic, in which the attachment figures/caregivers who are supposed to protect and care for the individual are at the same time a source of anxiety, threat, neglect and/or abuse. The effects of trauma, including childhood trauma, may translate into diagnoses of posttraumatic stress disorder (PTSD) or, as recently defined in the ICD-11, complex PTSD (CPTSD, Maercker et al., 2022), although not all patients with trauma histories receive these diagnoses. The PTSD diagnosis centers around persistent intrusive mental experiences related to, and mental and behavioral avoidance of, triggers and reminders of the event, along with alterations in cognitions and mood and hyperarousal (American Psychiatric Association, 2013). In the CPTSD diagnosis, these are combined with disturbances of self- organization, problematic interpersonal relationships, and affective dysregulation (World Health Organization, 2019).

There is a substantial overlap between these (C)PTSD diagnoses and personality disorder diagnoses, especially borderline personality disorder (BPD), which is frequently linked to early adversity (Zanarini and Frankenburg, 1997; Ford and Courtois, 2021). The prevalence of CPTSD is estimated at about 36% in adult clinical populations, rising to 50% among patients with BPD (Møller et al., 2020; Ford and Courtois, 2021; Maercker et al., 2022). Similarly, PTSD prevalence in BPD patients varies between 30 and 50% in community and clinical samples, respectively (Zanarini et al., 1998; Grant et al., 2008; Pagura et al., 2010; van Dijke et al., 2018; Møller et al., 2020). These three diagnostic categories, while sharing symptoms and etiological factors, can be differentiated both empirically and phenomenologically and might represent a spectrum of posttraumatic syndromes (Ford and Courtois, 2021). This spectrum starts with traumatic victimization, evolving into more severe conditions from PTSD to CPTSD (characterized by disturbances in self and relational functioning) and eventually to concurrent CPTSD/BPD. Such a latent severity dimension underlying the distinct diagnostic categories is paralleled by evidence that patients with co-occurring BPD and PTSD exhibit lower quality of life, more severe BPD symptoms, increased dissociative symptoms and comorbidities, higher suicide attempt rates, more frequent childhood trauma, and greater feelings of worthlessness compared to patients with only one diagnosis (Pagura et al., 2010; Bateman et al., 2023c).

From a treatment perspective, the complex co-occurrence of disorders following trauma has been acknowledged in programs targeting personality disorders and trauma-related conditions. However, approaches focusing on trauma and those addressing personality disorders have evolved separately. While certain patients benefit from existing treatments for trauma or personality disorders, a notable gap exists between these modalities. Early evidence suggests that in treatments like mentalization-based treatment (MBT) and dialectical behavior therapy (DBT) for BPD, patients with concurrent PTSD symptoms often exhibit more severe symptoms and worse outcomes (Barnicot and Crawford, 2018). Particularly, BPD patients with significant childhood trauma respond better to more intensive treatment, which signifies the challenge trauma introduces in treating personality disorders (Smits et al., 2022). For patients with co-occurring (C)PTSD, BPD treatments may lack an adequate focus on trauma symptoms, pointing to the necessity for tailored interventions that tackle trauma sequalae, including dissociative symptoms (Shah et al., 2020; Rüfenacht et al., 2023b). Conversely, in PTSD treatments, patients with comorbid personality disorders find benefit but face poorer outcomes compared to those without such comorbidities (Slotema et al., 2020; Snoek et al., 2020). Additionally, current PTSD treatments may not effectively address CPTSD (Maercker et al., 2022). With evolving clinical guidelines for CPTSD, treatment recommendations now include multi-component interventions focusing on safety, psychoeducation, collaborative care, and strategies for self-regulation, distress tolerance, and trauma-specific methods (Maercker et al., 2022). Hence, observations from the field of trauma-focused interventions also underscore the need for tailored treatments that alleviate persistent difficulties in self and relational functioning, regardless of the diagnoses of personality disorder or (C)PTSD.

Such treatments are rare, although efforts to integrate a trauma focus within personality disorder therapies, and vice versa, are emerging. For instance, DBT-PTSD, a version of DBT integrating prolonged exposure, is effective for patients with BPD and PTSD (Bohus et al., 2019, 2020). Patients who complete DBT-PTSD showed significant and more lasting improvements in PTSD symptoms, along with reduced suicide attempts, self-harm, dissociation, trauma-related guilt, and enhanced overall functioning compared with those receiving standard DBT (Harned et al., 2018a,b). Notably, these benefits were apparent only after reducing PTSD symptoms and cognitive issues, with improvements in PTSD following the start of trauma memory processing. However, high dropout rates occurred before this processing began, and DBT-PTSD did not outperform standard DBT in reducing interpersonal problems. Still, the initial results of adaptations like DBT-PTSD are promising, advocating for further refinement of treatments. This aligns with recommendations for a flexible, modular-based approach that can be tailored to each patient’s needs (Karatzias and Cloitre, 2019).

Similarly, MBT has placed increasing emphasis on directly addressing trauma (Luyten and Fonagy, 2019; Luyten et al., 2020b), leading to the creation of trauma-focused mentalization-based treatment (MBT-TF; Bateman and Fonagy, 2021; Bateman et al., 2023a). We have always assumed that trauma impairs mentalizing, and limitations of mentalizing account for some trauma-related symptoms, such as flashbacks and dissociation (Allen and Fonagy, 2010, 2019). The merit of mentalizing interventions to address the impact of trauma is supported by evidence linking adversity to ineffective mentalizing (Wagner-Skacel et al., 2022), along with studies evidencing the mediating impact of mentalizing and epistemic trust in the relationship between adversity and trauma-related symptoms, such as dissociation or relational difficulties (Hayden et al., 2019; Kampling et al., 2022; Bateman et al., 2023c). Moreover, preliminary evidence supports the notion that improvements in epistemic trust positively impact treatment outcome for CPTSD (Lampe et al., 2024).

Even though traditional MBT has been shown to be quite effective for patients with a history of (complex) trauma (Smits et al., 2022), these patients often have considerable difficulties engaging in the treatment, especially in the early phases, due to their avoidance strategies. This negatively impacts their own treatment process, potentially leading to stagnation or dropout, and can also affect the engagement and treatment process of other patients. Therefore, to optimize treatment outcomes, there was a need for a more explicit focus on trauma and its consequences within MBT.

In keeping with other models of trauma treatment, MBT-TF follows a phased approach to treatment (Herman, 1998). MBT-TF differs from traditional MBT in this more specifically phased approach, its (treatment and sessional) structure, and the more explicit focus on trauma processing. It is based on our notion that for patients significantly impacted by (complex) trauma, merely enhancing general mentalizing abilities may not adequately improve an individual’s capacity to manage trauma memories and their impact. MBT-TF, therefore, explicitly addresses the ineffective mentalizing of traumatic experiences and the consequences for self- and other-representations and relational functioning. To attain this, MBT-TF highlights the role of trauma symptoms and their consequences during the assessment phase, placing them at the centre of a co-created trauma-informed formulation of the patient’s functioning. MBT-TF places an even greater emphasis than traditional MBT on establishing shared group norms and values at the onset of treatment, to promote safety and reduce the need for mental and social isolation. In this way, avoidance behaviors, which in traditional MBT tend to disrupt the treatment process, are mitigated. Moreover, particularly in the second phase, MBT-TF sessions are more structured than traditional MBT group sessions, facilitating the sharing and processing of traumatic memories and providing the necessary emotional scaffolding. Finally, in the ending phase, MBT-TF explicitly focuses on mourning and the loss caused by trauma in the patients’ lives. Overall, MBT-TF’s unwavering focus on improving trauma-focused mentalizing and promoting salutogenesis necessitates a process-oriented approach to intervention, facilitated by an experienced team of healthcare professionals, distinguishing this approach from peer support groups.

Unlike most trauma treatments, MBT-TF is delivered in a group setting, utilizing group therapy as a means to recalibrate the traumatized mind, which is often mired in shame and isolation, hindering recovery (Leskela et al., 2002; Øktedalen et al., 2015; Stotz et al., 2015; Schomerus et al., 2021). Although evidence supporting the effectiveness of group treatment for PTSD is accumulating (Sloan et al., 2013; Schwartze et al., 2019; Griffin et al., 2023), group trauma treatment is still underrepresented in treatment guidelines, and empirical studies on group interventions targeting CPTSD are scarce. Yet, from a mentalizing perspective on trauma, a group-based approach may be helpful as it provides an optimal context to foster social connection within a mentalizing framework, which is assumed to be crucial for mitigating trauma’s detrimental effects on self and relational representations and dynamics.

This paper is the first to comprehensively outline the rationale, development, and core principles of MBT-TF, along with a detailed clinical illustration based on our 2 years of accumulated experience with the model. We first summarize the mentalizing perspective on trauma as a basis for understanding the presumed change mechanism and core principles of MBT-TF. Subsequently, we present the clinical approach, covering the treatment structure, phases, key principles, interventions, and common challenges, illustrated through the clinical vignette of Ellen.

## Context: a mentalizing approach to trauma

Emerging research indicates that trauma, especially complex trauma, disrupts three central capacities vital to the development of psychopathology, alongside severe dysregulation of the stress system (Luyten et al., 2020a,b; Nolte et al., 2023): (a) the ability to form healthy attachment relationships; (b) mentalizing, that is, the capacity to understand to understand oneself and others in terms of intentional mental states such as needs, desires, feelings, beliefs, and goals (Allen and Fonagy, 2006; Allen et al., 2008); and (c) epistemic trust, or the ability to accurately identify specific others as trustworthy and, therefore, be able to adequately rely on the information they convey as personally relevant and generalizable, and by that means, the individual’s capacity to accept and internalize new information; hence the addition of the descriptor ‘epistemic’ to indicate a specific element of general trust in others (Fonagy et al., 2015, 2019).^1^

In typical development, the attachment system activates in response to increased arousal or threat, leading individuals to seek closeness to responsive attachment figures, thereby reducing distress (Bowlby, 1973, 1988; Mikulincer and Shaver, 2007). Attuned parenting, marked by significant affect mirroring and the use of clear cues (e.g., eye contact, motherese), fosters attachment security and, in turn, the development of epistemic trust in children, meaning trust that the parent is a reliable source of knowledge about the internal and external world (Fonagy et al., 2007; Fonagy and Luyten, 2018). This trust is essential for the unencumbered expression of the innate capacity to learn through social interactions and is linked with resilience and salutogenesis, that is, the ability to benefit from others’ positive influences (Antonovsky and Sagy, 1986) and their co-mentalizing. Being recognized and mentalized within this attachment relationship helps regulate arousal, develop secondary representations of self-states and exercise effortful control, ultimately fostering broader mentalizing abilities (Fonagy and Allison, 2023; Nolte et al., 2023). However, this adaptive cycle is disrupted by stress and adversity, with trauma particularly impacting the development of epistemic trust and mentalizing abilities. Complex trauma often places individuals in a paradoxical situation where caregivers, expected to provide comfort and reduce distress, are also sources of severe conflict, abuse, or neglect (Teicher and Samson, 2013), leading to a defensive suppression of mentalizing to protect against the painful perspective of the abuser.

As a result, individuals might excessively use hyperactivating or deactivating secondary attachment strategies^2^ to adapt to environments marked by inconsistent, unresponsive, or abusive figures (Ein-Dor et al., 2010; Ellis et al., 2011; Luyten et al., 2021). These environments may also cultivate high levels of epistemic mistrust and vigilance as adaptations to perceived malintent or mistreatment, or conversely, engender epistemic naivety due to the misjudging of trustworthiness resulting from erroneous filtering of what can, and what cannot, be trusted (Luyten et al., 2020a). This situation is linked with an increased risk of disrupted self-other boundaries, distorted secondary representations, a fragmented and depleted self-concept, and overall impaired mentalizing and affect regulation abilities generally (Fonagy et al., 2002, 2010; Fonagy et al., 2017). Empirical evidence shows that insecure and disorganized attachment patterns, often associated with adverse childhood experiences, mediate trauma symptoms (Liotti, 2006; MacDonald et al., 2008; Byun et al., 2016; Luyten et al., 2020b). Moreover, recent studies highlight that mentalizing mediates the effect of attachment on interpersonal distress (Hayden et al., 2019). Following this, we recognize that ineffective mentalizing can make an individual particularly vulnerable as it exaggerates the negative consequences of adversity.

**Figure 1:**
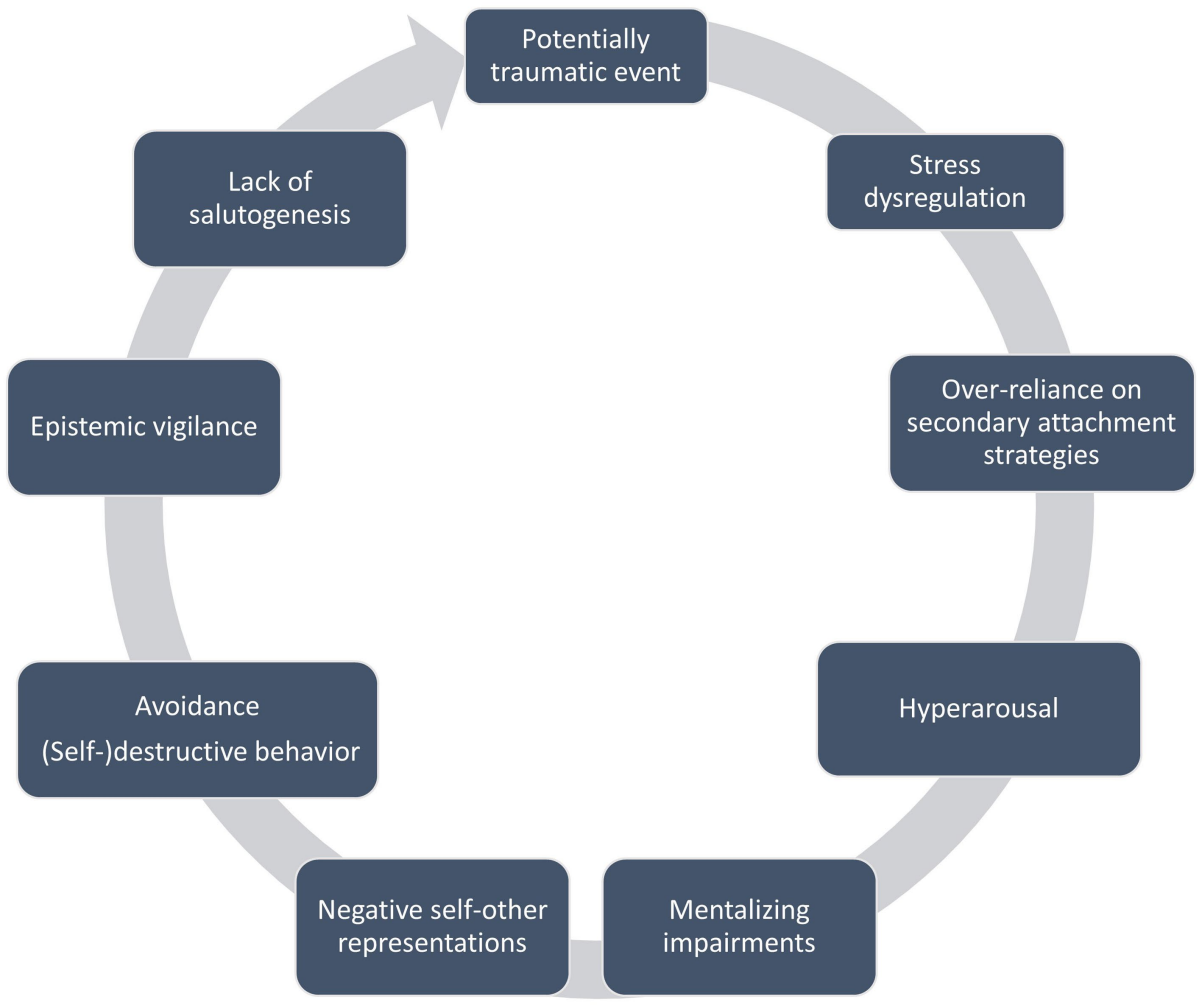
The interplay between trauma, attachment, mentalizing, and epistemic trust.

The interplay between trauma, attachment, mentalizing, and epistemic trust is complex and reciprocal (see Figure 1). The lack of co-regulation and the opportunity to recalibrate the traumatized mind within a secure mentalizing attachment relationship detrimentally affects both the ability to mentalize effectively and the concurrent development of epistemic trust. Furthermore, deficiencies in attachment, epistemic trust, and mentalizing may in turn also exacerbate the impact of trauma on an individual’s experience and functionality, as early adversity leads to an over-sensitized attachment system and heightened vulnerability to stress. This increases the likelihood of future adversities, especially in interpersonal contexts. Without stress co-regulation, individuals often remain in a state of heightened arousal and vigilance to perceived threats, resulting in cognitive difficulties, irritability, and aggression as they persist in fight, flight, or freeze responses. Additionally, reliance on secondary attachment strategies can be detrimental over time, trapping individuals in the belief that others are ultimately unavailable to provide care and support. This misperception intensifies the reliance on ineffective modes of mentalizing and (self-) destructive behaviors to shield against overwhelming feelings of anxiety, anger, shame, guilt, and a disintegrated sense of self.

Trauma also typically results in an unstable self-concept and a disjointed, distorted self-narrative, influenced by ‘alien-self’ experiences; such experiences are conceptualized as involving the internalization of an abusive caregiver’s or perpetrator’s perceptions or attributed thoughts, defining the self with these painful, unmentalized aspects (Bateman and Fonagy, 2021), e.g. “I am shameful and should be ashamed”. These alien-self experiences often lead to self-destructive acts as the traumatised individuals attempt to gain control of an internalized abusive figure, who they experience as hurting them from within, by externalisation and projection. Such efforts then lead to interpersonal conflicts, often triggered by reminders of trauma, intensifying feelings of shame, guilt, or worthlessness, and fostering destructive behavioral patterns. These re-enactment cycles, that is, patterns of interpersonal interaction used to manage trauma symptoms that particularly resemble the traumatic relational patterns from the past and are repeated in current interactions, contribute to high levels of revictimization (Cloitre et al., 1997; Widom, 1999) and the intergenerational transmission of trauma and psychopathology (Berthelot et al., 2019).

The bidirectional impact of these processes implies that ineffective co-regulation and mentalizing of traumatic events and its effects perpetuate distress, leading to breakdowns in mentalizing in which prementalizing modes^3^ dominate functioning (Allen and Fonagy, 2010; Luyten et al., 2020a). Conversely, when experienced in prementalizing modes, the emotional re-experiencing of trauma may in turn feel more immediate and destabilizing, such as flashbacks in the psychic equivalence mode of ‘inside-out’ thinking when subjective experience is felt to be equivalent to external events. The intensity of unmentalized experiences may prompt avoidance strategies through dissociation in pretend mode or may instigate physical, and sometimes (self-) destructive, actions to cope with unbearable self-states from a teleological perspective. Unmentalized, distorted perceptions of others that foster relational mistrust further sever social connections, contributing to mental and social isolation. The associated emotional experience of shame, often linked with trauma, obstructs resilience through social referencing and help-seeking. Consequently, individuals may erect barriers of mistrust and social vigilance, avoiding potentially beneficial social interactions and the opportunity for positive social feedback (Campbell et al., 2021; Kampling et al., 2022; Nolte et al., 2023) that would contradict their trauma-influenced perceptions. Ironically, even when exposed to alternative, constructive reflections of themselves, individuals may struggle to accept these perspectives due to their epistemic mistrust and the lack of resonance of this more benign mirroring with their entrenched negative self-views and perceptions of others shaped by trauma. It is as though individuals not only lack the internal mechanisms to steer clear of harmful experiences but also evade the social referencing needed to adjust their internal compass.

In summary, from a perspective focused on mentalizing, we suggest that trauma instigates a sense of epistemic dysfunction, a distrust in the reliability and trustworthiness of the world. This lack of trust significantly hinders an individual in social settings, as they miss the opportunity to learn and sustainably adjust their beliefs and feelings through positive social interactions and experiences. As a result, the traumatic experience remains isolated, lacking social context, which perpetuates distorted thoughts and emotions, such as shame and guilt. Following this, we propose that re-establishing social connections within a context that emphasizes mentalizing can effectively counteract the widespread negative effects of trauma on both self-perception and relationships and alleviate shame and isolation. We assume this process of reconnection and shared understanding of experiences to be crucial for the recalibration of the traumatized mind and for interrupting the cycle of harmful self- and other views generated by trauma that instigates vicious patterns of interaction that are harmful and potentially self-perpetuating.

## Key principles and mechanisms of change in MBT-TF

MBT-TF, rooted in the mentalizing framework for understanding trauma, posits that the traumatic effects of adversity stem not solely from the event itself but more so from the isolation of the individual’s mind and the experience of enduring these overwhelming experiences alone, without another mind to help buffer the emotional intensity and to assist in making sense of it through social referencing. Therefore, MBT-TF focuses on four main goals: (1) improving mentalizing related to the trauma, (2) reducing psychological isolation, (3) decreasing social vigilance, and (4) alleviating shame, thereby also fostering the potential for epistemic trust and facilitating social referencing (Bateman and Fonagy, 2021).

Critical to the understanding of the principles of MBT-TF is our notion that merely enhancing general mentalizing abilities may not directly improve an individual’s capacity to manage trauma memories and their impact. Rather, concentrating on the ineffective mentalization of traumatic experiences is likely to strengthen a more generalized mentalizing process that gradually extends into extra-therapeutic relationships. Designed as a group intervention, MBT-TF emphasizes the processing of specific trauma memories through sharing and collectively mentalizing these experiences as a shared aim between all participants. Discussing and processing trauma memories in a group setting is crucial, as patients often try to avoid (talking with others about) these memories, seeking to block them out due to the debilitating shame and overwhelming emotions they elicit. However, such avoidance keeps trauma experiences unmentalized, isolated, and frozen in time, leading to the activation of these memory fragments in certain contexts (causing symptoms such as intrusions, flashbacks, dissociation). As this tendency is shared but is deployed in a context specific to each member of the group, collective processing of trauma related thoughts and feelings benefits each traumatized individual.

Trauma processing sessions in MBT-TF, where the trauma narrative is shared and reflected upon in a group, are not intended as mere exposure or desensitization. Instead, the focus is on fostering a mentalizing process around the traumatic experience, expressed within a framework that allows social referencing of the experience, aiming to integrate the memory as a mentalized and reflected-upon experience. This involves activating all aspects of memory—autobiographical, semantic, as well as procedural and emotional, implicit memory—that encapsulate coping mechanisms and the general views the individual has about self and others related to the traumatic event. Beyond mentalizing the trauma itself, MBT-TF addresses the impact of trauma on self and relational functioning, which is crucial for breaking the repetitive cycles of re-enactment prevalent in patients’ lives. Sharing and exploring the impact trauma has had on thoughts and affects about oneself in relation to these events, and then hearing others’ perceptions and understandings of the individual, offers a chance for ‘recalibration’ through the understanding of others, particularly those more likely to be trusted because of shared experiences. Sharing reduces isolation and shame and helps modify both self- and other representations through the actual experience of interpersonal interaction, potentially enabling a more profound and sustained change than mere cognitive reappraisal.

Developing a collective understanding of how trauma affects current functioning, from ‘we-mode’ or a shared perspective^4^, restores a sense of belonging and agency, and provides a context for revising self and other representations (Gallotti and Frith, 2013; Fonagy et al., 2022; Bateman et al., 2023b). Witnessing and listening to others’ experiences helps diminish shame and enhances the mentalization of previously unprocessed traumatic content. Genuine interactions with others, when emotional arousal is well managed, allow for new social experiences, which, by influencing self and other representations, reduce epistemic mistrust and vigilance or inadequate credulity. Encountering empathy and compassion from individuals with similar traumatic backgrounds, and hearing them express their challenges and its impact on their current lives, prompts a dissonance that encourages patients to recognize and question social actions that come from alien self-experiences. Listening to others share and reflect on their experiences indirectly aids in gradually adjusting distorted self-views. Moreover, the group setting offers a safe environment for social learning and positive exchanges among patients.

Given the profound effects of trauma on stress regulation, MBT-TF places a special emphasis on embodied mentalizing. MBT-TF addresses explicitly the failed interoception as a key aspect of ineffective mentalizing brought about by a traumatized mental state, by focusing on bodily sensations and connecting them to mental states. This approach is particularly critical for individuals whose bodies are sites of trauma, such as in cases of sexual or physical abuse, where dissociation and a complete disregard for bodily symptoms or avoidance of internal experiences have become survival strategies due to feeling unsafe in their own bodies.

MBT-TF is structured into three phases, aligning with established recommendations for trauma treatment. The first phase includes psychoeducation about mentalizing, trauma, and strategies for managing intense emotions and dissociation, aiming at symptom stabilization and installing safety along with promoting epistemic trust. The second phase is dedicated to processing specific traumatic memories. The third phase deals with grief, acceptance, and focuses on moving forward. These phases correspond to the three three distinct processes of communication, as conceptualized within the mentalizing framework, that are assumed to cumulatively account for change in psychotherapeutic treatments (Fonagy et al., 2019; Luyten et al., 2020a):

1. *Communication System 1* focuses on establishing epistemic trust and creating an ‘epistemic match’^5^ in a secure, low-arousal environment. The therapist provides a model for understanding the mind that aligns with the patient’s experiences, promoting recognition and comprehension.
2. *Communication System 2* emphasizes the re-emergence of mentalizing, and is pivotal in MBT-TF for processing traumatic memories. As patients become more receptive to social communication, the therapist and patient engage in a collaborative process of understanding and integration. This is characterized by a mutual genuine interest and curiosity about their own minds and those of others, reinforcing and building upon epistemic trust. This, in turn, initiates a virtuous cycle where enhanced and balanced mentalizing facilitates more meaningful engagement with social information and networks.
3. *Communication System 3* focuses on applying social learning to broader contexts. It underscores the importance of extending therapeutic achievements—namely, the restoration of epistemic trust and improved mentalizing abilities—into the patients’ lives beyond the treatment setting. In MBT-TF, this involves specifically addressing grief, acceptance, and the process of moving forward to be able to orientate towards the social world with all its benefits, central among which is the deposit of accumulating human understanding: culture. Although not often talked about in the context of psychotherapy, being deprived of access to shared social knowledge is a central problem of the traumatised individual. Therapy works when a traumatised person reconnects with the collaborative process of learning and teaching about how the world is—which is part of what being human is all about (Fonagy and Allison, 2023).

## Population

MBT-TF addresses the effects of complex trauma. Patients (1) report a history of complex traumatic experiences and (2) display a wide range of psychopathology, including (3) significant challenges in personality functioning manifesting as pervasive difficulties in identity, self, and relational functioning, which (4) often lead to destructive behavioral patterns. Additionally, they exhibit (5) enduring post-trauma symptoms such as hyperarousal, hypervigilance, intrusions, flashbacks, avoidance behaviors, and dissociative experiences. The exclusion criteria for MBT-TF are minimal. MBT-TF does not exclude patients who exhibit self-destructive behaviors, recognizing these behaviors as attempts to manage unprocessed intrusions or dissociative states caused by trauma. However, tailored interventions may be necessary for individuals who experience prolonged and severe dissociation (Rüfenacht et al., 2023b). Establishing a consensus on a collaboratively developed crisis plan is crucial at the onset of treatment. Current substance dependency might be considered a contraindication if more specific treatment for reduction of substance use is required.

Ellen, as depicted in Box 1, serves as a representative example of a typical patient whose progression through MBT-TF will be elucidated in what follows through clinical illustrations spanning the different stages of the therapeutic process.

BOX 1 **The case of Ellen**

Ellen, a 47-year-old woman, has been living alone since her divorce 2 years prior. She is a mother to a 22-year-old daughter and a 20-year-old son and works in a home for elderly people. Ellen’s principal challenges include difficulties in forming relationships, isolation, recurring depressive episodes, self-harm, mood instability, and struggles with daily life management. She is plagued by persistent painful memories, dissociative episodes, nightmares, and physical ailments. Ellen was directed to a MBT program designed to address BPD pathology. Additionally, MBT-TF was introduced as a supplementary modular program to her standard MBT regimen. Ellen, the daughter of mixed parentage, grew up with a younger brother in a strict religious community in Turkey, where social norms heavily influenced and dominated daily life. Ellen’s childhood was marked by maltreatment and neglect. She recalls her mother as perpetually anxious, fearing any misbehavior from her children. Both her parents were prone to adopting a highly accusatory stance when they perceived their children’s actions as deviating from social or religious norms, resulting in punishment. Her father, a dominant figure with a devout religious stance and substance dependency, occasionally exhibited violent behavior towards his wife and children. She recalls being witness to extreme violent inter-parental conflict. Ellen became an anxious child, overly cautious about making errors, and from a very young age felt compelled to manage and protect her younger brother. She remembers being scared all the time, ruminating a lot over what she had said or done to deserve punishment (being sent to her room, or having to write out and practice prayers for hours), convinced that she was at fault and a bad child. A pivotal moment came at 15 when her mother decided to relocate to her home country with her children, forcing Ellen to adapt to a new country and culture, a transition fraught with challenges. At school, Ellen often felt singled out, reinforcing her belief that there was something wrong with her. Being bullied at school instigated her way of handling fear by retreating into herself, trying to block out her feelings. Feelings of guilt were intensified by seeing her mother struggling to build a new life, for which she would blame Ellen and her brother during emotional outbursts. Although Ellen managed to finish her education, married, and raised two children, with whom she has relatively stable relationships, she experiences recurrent difficulties in relationships.

## MBT-TF’s clinical approach: treatment phases, foci and key principles

### Phase 1: stabilization, safety, epistemic match and shared formulation

This initial phase consists of a brief series of individual sessions, ranging from four to six sessions. As with other contemporary trauma treatment approaches, the emphasis at this stage is on ensuring safety and stabilization and, in MBT-TF, developing relationships with the therapist and group members, which helps to generate a safe group environment. Establishing a consensus on a jointly created crisis plan is critical at the treatment’s outset, as is stimulating patients’ motivation and commitment to work on trauma within a group setting. Patients are also encouraged to involve their significant others (attachment figures) in at least one individual session to educate them about the treatment approach, thereby enhancing support and facilitating the generalization of safety measures and therapeutic progress in the patient’s life outside (see Box 2). The practicality of including individuals from the patient’s external environment largely hinges on the positivity and suitability of the social context, as some clients may lack any supportive social contacts or have become so isolated that involving others in their treatment only becomes feasible as progress is made.

BOX 2 **Enhancing safety and grounding techniques in the early stages of Ellen’s treatment**

Ellen was eager yet apprehensive about her involvement in the MBT-TF program. To alleviate her concerns, therapists extended reassurance, support, and empathy, and provided an overview of the MBT-TF program’s objectives and structure, which helped reduce her anxiety. Ellen identified as treatment goals being able to manage dissociative symptoms and share about her traumatic memories, along with being able to manage overwhelming negative self-thoughts and mistrust towards others in a way that would reduce the need to withdraw herself and allow her to connect with others and alleviate her loneliness. In the initial assessment and engagement phase, discussions about her coping mechanisms included strategies to handle dissociation within the group setting. Ellen discovered that moving around helped mitigate the onset of dissociation, and she pinpointed three grounding techniques that were particularly beneficial. Additionally, she found solace in visualizing her dog, imagining being with her pet at home as a means to decrease arousal. Ellen, her therapist, and her group peers agreed to remain vigilant for signs of dissociation and to offer support as needed. During the individual sessions of MBT-TF, Ellen invited her adult son to attend a session with her. This provided Ellen an opportunity to discuss her traumatic past, much of which was previously undisclosed to her son. Although her son was initially shocked by these revelations, the disclosure facilitated a deeper understanding of Ellen’s behavior and its impact on their relationship, prompting him to offer his support. This process also allowed Ellen to reflect on her feelings of shame for not having shared her experiences sooner. The formulation of a crisis prevention plan was another critical step, with a particular focus on Ellen’s propensity to isolate herself when feeling threatened. Her son committed to recognizing and addressing this behavior by signalling to Ellen and actively reaching out during such times.

Individual sessions focus on psychoeducation and an individualized assessment of trauma’s impact on the patient’s life. With this, clinicians present a coherent model to help patients understand traumatized minds, the symptoms associated with trauma, and how treatment can address these issues. Conversely, therapists learn from patients, adapting the model to fit their unique narratives, thoughts, and feelings. This mutual educational and assessment process, characterized by a genuine, curiosity, the ‘inquisitive stance’ (Bateman et al., 2023b), treats the patient as an independent entity; being seen as capable of making decisions enhances their sense of being listened to, recognized, and potentially understood. This can support the re-establishment of epistemic trust.

**Figure 2:**
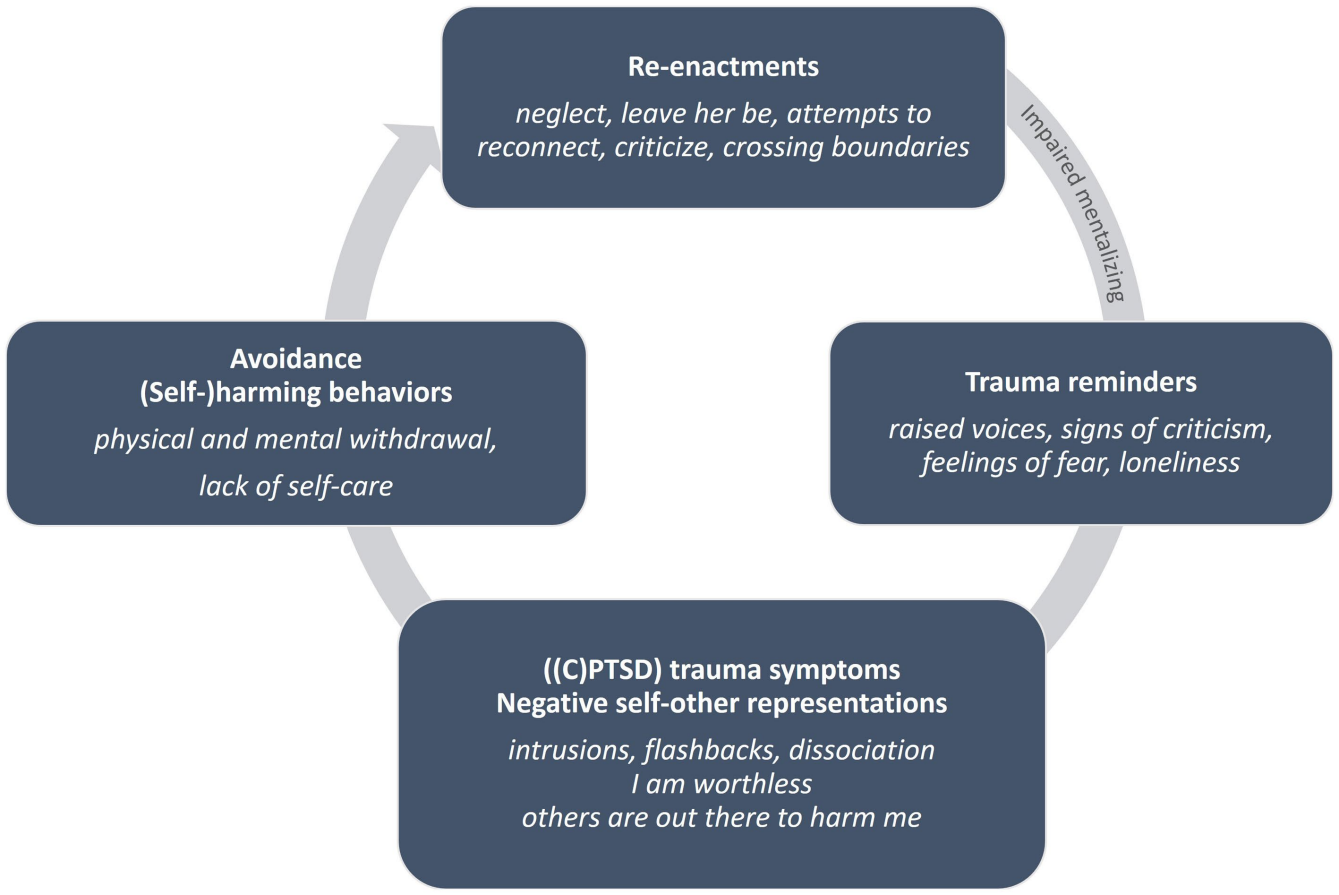
Ellen’s re-enactment cycle.

The initial assessment evaluates the patient’s mentalizing strengths and vulnerabilities, which is continued in momentary assessments throughout treatment, with the objective of continuously balancing the optimal level of arousal and tailoring interventions to the patient’s mentalizing capacity to avoid potential iatrogenic harm of re-traumatization. Assessment includes a comprehensive mental health review covering current symptoms, treatment history, context, and, crucially, resilience factors, with a particular emphasis on trauma history, triggers, and the identification of the most pressing trauma symptoms experienced by the patient. These symptoms include (1) intrusions, flashbacks, nightmares; (2) anxiety and dissociative symptoms linked to trauma triggers connecting current to past experiences; (3) trauma-related emotions such as shame or feelings of being ‘dirty’; (4) self-perception issues like self-criticism, negative self-image, and alien-self experiences of ‘badness’; and (5) avoidance strategies that in turn potentially exacerbate trauma effects, as a result of detachment from internal experiences including physical, emotional, and mental intimacy. A relational map and attachment style assessment help visualize and explore trauma’s effects on current relational functioning and identify interpersonal difficulties contributing to trauma re-enactment cycles (see Figure 2 and Box 4 for an illustration of Ellen’s re-enactment cycle). The aim is to develop a shared initial understanding between therapist and patient regarding the patient’s current challenges in relation to their traumatic experiences and their effects on self, others, and their broader world interactions (Bateman and Fonagy, 2021). This is more than solely the patient’s experience or the clinician’s understanding of that experience. This mutual understanding is translated into a shared formulation, which entails a mutually construed picture of a reality that is now shared between patient and clinician and available to be explored jointly.

The MBT-TF formulation (see Box 3 for Ellen’s initial MBT-TF formulation) follows the key principles of the formulation as described in the generic model of MBT (Bateman et al., 2023b). First, the structure and format of a formulation is tailored to the preferences of the patient. It is for the patient; it is not to show the depth of the clinician’s psychological understanding. Many are done in written form (often the shorter the better) but an equal number are done in diagrammatic form. Second, the central reference point is mentalizing and its vulnerabilities. Third, it is collaborative and jointly generated. Fourth, it has to be understandable to the patient and stimulate a feeling and representation in them of the clinician recognizing them (“I am with someone who is seeing me as I see myself”). Fifth, it is dynamic and changes as treatment evolves, and is reviewed and re-written at regular intervals. The patient uses the brief formulation (relational passport) to introduce themselves to the group and it is used subsequently throughout group sessions. Identifying trauma reminders or triggers, which can reactivate negative or self-destructive responses, enables patients, therapists, and group members to recognize potential re-enactments as they may occur within group sessions. The development of this formulation is often complex given trauma’s typical impact on memory coherence and patients’ challenges in articulating their experiences. The formulation thus remains tentative, requiring adjustments as understanding deepens. Emphasizing the exploration of the internal world over the formulation ‘product’ is a key goal.

BOX 3 **Ellen’s initial MBT-TF formulation**

We have discussed your childhood and the difficult circumstances you experienced. It is unsurprising that you ended up becoming frightened and easily startled by others and do not trust anyone. Your independence and trustworthiness were key strengths that helped you to build a life for yourself and raise your two children, who are doing well, which you can be proud of! Also, at work, you manage to overcome arousing and threatful situations, which is not easy but very important to you, as taking care of the elderly provides you with the sense of belonginess and connectedness that you often deprive yourself of but can long for as well. When you are with others, you are often scared and you mostly try to hide from others. You also rightly try to keep your past out of your mind most of the time, so you can function. When the past does come to your mind (in flashback memories or nightmares) you panic and cannot think. We have called that ‘blow-up time’ and we need to watch out for it – when it occurs and if it occurs in treatment (*psychic equivalence*). Do not forget, the first thing to do to get mentalizing back is to calm down, so we will work on that first and have also agreed that your son will try to help you when this happens at home.

**Impact of traumatic events**

Between us we decided that you will talk about a violent episode between your father and mother and try to express what you experienced when it was happening. You identified one particular memory of an incident in which your father was violent towards your mother when you were 10 years old, and you and your brother hid upstairs. You tried to protect your brother from witnessing the violence, but could not prohibit him from running down the stairs, after which he got hurt by your father as well. For you, this memory captures the violent atmosphere you grew up in, as well as the feelings of fear, shame and guilt that you felt and still often experience. In sharing this episode in the group, you will try to listen to others’ responses as they try to help you express the experience.

**Relationship to yourself and others**

We have talked about how you think about yourself (*I-mode*). Much of the time, you experience yourself as worthless and shameful and blame yourself for not protecting your brother from the violence and fear. Listening to others is hard for you as you constantly imagine that they are against you or might want to hurt you (*Me-mode personalized*). This might make you anxious in the group and mean that it is difficult to consider what others say. You tend to care for others and might need to watch out for this in the group when others present (*Me-mode*). Hiding from others is your way of making it safe for yourself, with the downside of leaving you feeling alone and separated from supportive social interaction. As this might happen in the group as well, it is important to let the others in the group know about this. We will try to work together to keep you from completely distancing from yourself and others.

**Hopes/goals/aims for the group**

Change is possible if you can ‘borrow’ others’ minds to reconsider yourself. This is what the group is for (*generation of We-mode experience*). You want to be able to confront and face painful feelings without dissociating, and try to allow people to get to know you and connect with them.

Note: The clinician and Ellen draft a shared MBT-TF formulation during the initial sessions. This formulation is inherently dynamic, changed and rewritten at regular intervals as treatment evolves. Ellen used this formulation as a basis to formulate a *relational passport*, summarizing in her own words why she attends the group, with a focus on the relational challenges she experiences. The italic text in brackets refers to mentalizing processes for clarification and may or may not be discussed (in other terminology) with the patient.

Identifying a distressing memory closely linked to current trauma symptoms for further exploration in phase 2 group processing sessions is a further critical objective of the individual preparatory sessions. Pinpointing a specific memory can be a challenging task for individuals burdened with multiple, interconnected traumatic events.

In the therapeutic approach and clinical emphasis of MBT-TF, fostering the renewal of epistemic trust is prioritized in the initial phase, although reigniting social learning is recognized as a key mechanism of change throughout all later stages of therapy. Therapists should remain attuned to any forms of avoidance or withdrawal, especially during the early stages of treatment and the transition into group sessions, as is customary in MBT. Avoidance or withdrawal might not always be evident through physical absence but can occur at a level where there is resistance to accepting and assimilating insights from others. Participants may seem to listen attentively to feedback yet not exhibit expected behavioral changes. In MBT-TF, therapists must be particularly vigilant in maintaining a balance between activating the attachment system and the tendency to revert to survival mechanisms in reaction to trauma triggers associated with social or interpersonal contexts. These triggers can lead to heightened (fight/flight) responses or reduced arousal (dissociation), moving the individual outside their window of tolerance and obstructing mentalizing abilities. This sensitive balancing act is a critical aspect of why MBT-TF, as a group intervention, poses challenges but is instrumental in disrupting maladaptive cycles and fostering new social experiences, which in turn enable self-reflection.

After the preparatory phase 1, individual sessions may be offered according to patient need and depending on the context. The holding environment of treatment is an important anchor to provide safety. When needed, therapists offer follow-up calls or reach out to follow-up on commitment issues. Importantly, MBT-TF’s presumed working mechanisms focus on group work.

#### Initial group sessions: building relationships, ensuring safety and psycho-education

In the early group sessions, the main objectives of phase 1, of establishing safety in the group and developing epistemic trust, are prioritized in order to facilitate the trauma processing in phase 2. Patients get to know each other and the two facilitating therapists, and work together to create group norms and values (such as fairness, mutuality, confidentiality, kindness, open communication, respect, having a shared purpose centered on processing of trauma experience and the aim of mentalizing one another). Developing shared values is the first step to creating a reality in the group that is jointly owned, which can be referred back to by the group facilitators or patients when safety or group cohesion is challenged. The individual sessions of phase 1 help prepare the patients to introduce themselves to others. Initial group sessions are to match the individual presentations with each other to create a collective identity within the group and a shared purpose, both of which form part of ‘we-mode’ mentalizing. Therapists use exercises designed to enhance safety and regulate arousal, which vary in nature—some are more interactive and playful, while others focus on bodily awareness. These activities are particularly beneficial for patients who dissociate frequently, allowing them to feel part of the group without the pressure of having to share personal information or concentrate on potentially destabilizing topics. However, it is crucial to monitor for trauma responses that these exercises might inadvertently trigger (i.e., by increasing awareness or the perceived pressure to perform), necessitating careful observation and management by the clinicians of arousal levels in the group as a whole and in individual patients. Successful navigation of these moments of movement outside a ‘window of tolerance’ enables group members to start to notice and recognize when this occurs, giving the opportunity to rewind and learn how to manage it without collapsing into fight and flight or avoidance. Jointly focusing on the anxieties as they happen increases mentalized affectivity, that is, it gives context and meaning to the experience while remaining in the momentary emotion, which stimulates a mentalizing understanding of their reactions without directly confronting traumatic content.

The group’s focus then shifts towards collective psychoeducation, covering topics like (a) the impact of trauma on body and mind, (b) the window of tolerance of anxiety and its benefits for mentalizing effectively, (c) epistemic trust, (d) emotion regulation strategies (particularly for anxiety, shame, and avoidance); (e) understanding and managing common symptoms such as dissociation, flashbacks, and nightmares; (f) mentalizing versus prementalizing states, and (g) the value of social learning. Therapists clearly outline the treatment structure, furthering the psychoeducational goal and fostering interpersonal connections within the group. Collective psychoeducation is helpful in creating a shared culture for the group, contributing to it being a safe place to learn. This first phase also focuses on learning to identify and manage anxiety in a group setting. By sharing their personal formulations, patients learn about each other’s trauma triggers and the persistence of the effects of trauma in current relationships through cycles of re-enactment. Discussions of trauma histories are deliberately avoided at this point. Reflections on the experience of anxiety, particularly how trauma-related anxiety impacts current functioning, are encouraged. Patients are urged to be attentive to bodily sensations, which can become focal points at the beginning and at the end of each group session, helping them begin to link physical states to arousal and emotional states. The objective is for group members to recognize bodily sensations within themselves and observe subtle changes in others, fostering curiosity about the mental states these changes indicate, thereby enhancing embodied mentalizing. This approach requires openness and sensitivity, as it may provoke feelings of shame or activate avoidance strategies, which can, however, then also become topics for joint mentalizing.

The group collectively holds responsibility for being attuned to the re-emergence of trauma symptoms and re-enactment cycles (see Box 4). A continuous joint focus on promoting safety and mentalizing prevents patients from re-experiencing trauma symptoms and re-enactment patterns in non-mentalizing modes, thereby avoiding iatrogenic harm and re-traumatization. Complete avoidance of re-enactments is not feasible; moreover, these experiences enable patients to gradually make sense of and move beyond states that initially seem insurmountable, cultivating new coping mechanisms, providing a context for new social experiences facilitated by the support patients provide towards each other. These new experiences of navigating complex emotions and interactions may later be used outside the group in patients’ own social contexts.

BOX 4 **Ellen’s re-enactment cycle**

Ellen’s abusive background has left her hypervigilant in current social interactions, anxious and fearful of others’ aggression. *Trauma reminders*, such as raised voices or (non-verbal) signs of criticism, trigger *trauma symptoms* such as intrusive thoughts or memories of her father’s violence and thoughts such as “the other is out to harm me.” Ellen’s *deep-seated belief* in her worthlessness and self-blame (“I am worthless”) and the *experience of the other* as malevolent fuels her suspicion towards others, anticipating harm or criticism from them. In response to feelings of self-blame and shame that dominate her experience, Ellen withdraws mentally and physically to manage her stress and arousal (*avoidance*), actively trying to avoid thinking and feeling and avoiding close contact with others. This, in turn, causes others to perceive Ellen as distant, instigating interactions that particularly resemble the traumatic relational patterns from her past (*re-enactments*). Others react by distancing themselves, instigating the emotional neglect Ellen experienced as a young child, or conversely, try to re-connect with her in ways that Ellen experiences as intrusive, for example, by raising their voices or making physical gestures in attempts to engage her, which in turn increase Ellen’s need to distance herself. Arousal can get so extreme that she frequently dissociates. For example, when they were at a young age, Ellen’s children would persistently physically try to get her attention, which would arouse her even more when she was in an anxious mental state, causing her to dissociate. Also, in group therapy, therapists could overwhelm Ellen with questions, instigating more internal chaos and anxiety. During the assessment phase, Ellen and her therapist addressed this cycle (see also Figure 2) and its negative impact on her friendships, jobs and, potentially, the treatment process. Over time, Ellen—with the help of therapists and group members—became increasingly aware when this cycle was activated.

At the conclusion of phase 1, a group review session (see Box 5) allows members to reflect on their thoughts and feelings about the group work thus far, setting the stage for phase 2. Clinically, determining when the group is sufficiently safe to move to phase 2 may not be obvious and can be challenging. Regardless of whether the transition between phases is fixed by the number of sessions or remains flexible, clinicians often express concerns about progressing to phase two due to interpersonal challenges in the group. These challenges often stem from the live re-enactment of trauma cycles in interactions among group members, which can complicate group dynamics. Common issues include feelings of being different, singled out, isolated, disliked, or experiencing a sense of being targeted, bullied, or abused. Such experiences may echo past traumas but can also evolve into a significant belief system within the group’s dynamics, leading to conflicts among members or with facilitators. These tensions and anxieties within the group are actively addressed and managed in the context of psychoeducation and require attention when they occur in order to ensure they are addressed with a mentalizing stance. Importantly, therapists should avoid excessive delays in moving to the trauma processing of phase 2 and adhere to the agreed structure as much as possible, to prevent the perpetuation of avoidance behaviors.

BOX 5 **Review session following phase 1**

In Ellen’s group, the review session concluding phase 1 served to examine several key areas: the group’s effectiveness in collaboration, adherence to established group values, and resolution of some interpersonal issues. Members acknowledged their commitment and efforts and recognized the group’s challenges. Group members shared their anxieties and concerns about phase 2, many of which were shared between group members, which led to a feeling of mutual support and reciprocity. One member proposed instituting a check-in about how each person had experienced the group at each session’s end, a suggestion that received unanimous support and was incorporated into the group’s routine. During this session, Ellen expressed empathy towards other group members, noting that this mutual understanding helped her adopt a more empathetic view of herself.

When ready for the transition, information about phase 2 is revisited, and group members collaboratively decide the sequence of presentations for the trauma processing sessions in the next phase. This includes discussing the protocol if a member fails to attend their scheduled session. There is no prescribed answer and the group has to decide how to manage such eventualities. Patients are asked to commit to attending all groups in the second phase, using, when possible, a restatement of one of the main values of the group, e.g., mutuality and respect. Anxieties and shame related to sharing trauma experiences are discussed in the group setting, fostering curiosity among members about the similarities and differences in their experiences to help them join together around common themes, which stimulates we-mode mentalizing and promotes connection through a collective perspective. As an optional yet consistently applied approach, group members are encouraged to briefly share their life narratives at this point, preparing them to talk about personal experiences and help everyone begin to place each other in historical context.

Intermittent group review sessions, which can be reintroduced in phase 2 as necessary, offer a venue to address and repair relational disruptions, reaffirm the group’s norms and values, restore a collective mindset and rekindle we-mode, and facilitate reflection on how insights gained might apply to interpersonal dynamics outside the group.

### Phase 2: trauma processing—revitalizing mentalizing around traumatic memories

The second phase focuses on the processing of trauma memories identified by each participant during their individual preparatory sessions in phase 1. Group members proceed in a pre-agreed sequence with their processing sessions. It is recommended to organize these sessions into two rounds, allowing each participant to complete their first session before having a group review session of how the group is functioning, followed by a second round for all (see Boxes 6, 7). This structure ensures that all members benefit from the group’s increasing cohesion and provides a period for individuals to recover from the impact of the first session. When starting their second round, group members are invited to outline their experience of their first processing session and to reflect upon the personal take-home message formulated at the end of the first session. This approach allows for a richer experience in subsequent sessions, as social recalibration is reinforced in all sessions through (a) actively sharing as the presenter; (b) taking the role as listener to others sharing; (c) mentalizing in the group about how the group is responding to the presenter; and (d) discussing the influence of trauma processing sessions on external functioning and relationships. Trauma processing sessions are carefully organized and adhere to a stepwise procedure, which, in practice, may unfold non-linearly. The steps include:

1. *I-mode recall of the trauma narrative:* the patient designated to share is encouraged to recount their trauma narrative as openly as possible, supported by one of the group therapists, while the rest of the group and a co-therapist listen with minimal interruptions. Their task is to help the presenter express their narrative and not to comment on it in any judgmental way; the aim is to gradually expand on the trauma narrative, vividly invoking associated feelings to facilitate affective mentalizing of the trauma narrative (meta-cognition).
2. *Me-mode reflection on the trauma’s impact on themselves and their perceived experience of how others see them*: the group aids the patient in articulating feelings related to the trauma and how others see them as a person who has experienced such devastating events, both at the time of the event and currently. This collective reflection helps deepen their understanding of the trauma’s emotional aftermath through understanding the reflection of others (first-order mentalizing).
3. *We-mode joint meta-perspective:* this involves reflecting on the group’s shared understanding of the trauma narrative, considering both past and present perspectives.

BOX 6 **Ellen’s first trauma processing session**

Ellen selected a vivid traumatic memory involving her father’s abuse of her mother for her processing session. Initially, she was concerned that the memory might not be deemed severe enough, fearing judgment from others. The therapeutic environment, enhanced by group members’ empathetic responses, helped Ellen regulate her emotional response and engage in the processing work. As Ellen shared her story, the therapist’s inquiries into her location, her parents’ whereabouts, sensory details (what she saw, smelled, heard), and her bodily impressions and physical sensations helped her navigate through the fragmented and intrusive nature of her memory. A particularly distressing moment Ellen recalled was her attempt to hide upstairs with her younger brother, who, in his distress, ran downstairs and was also hurt. Sharing this incident evoked profound self-directed anger for not protecting her brother. Nonetheless, with the group’s support, Ellen articulated these intense feelings, weaving them into her narrative and shifting from distress to a moment of relief and brief pride. While Ellen shared her story and afterwards, group members responded supportively, prompting reflection on the group’s collective experience during the session. The therapists facilitated an exploration and validation of these varied experiences, carefully managing arousal levels. This dialogue and empathetic engagement, free from blame and emphasizing support, represented a shift from Ellen’s habitual patterns of re-enactment. Through these group interactions, Ellen experienced a sense of being understood, diminishing her self-critical views. A particular response from a group member, expressing concern over Ellen’s self-criticism (“You were 10 years old, just a child yourself, you were not to blame!”) initially introduced confusion for Ellen. This particular response offered a new, compassionate viewpoint, countering Ellen’s fears of being judged as inadequate for not protecting her brother against their parents. Past experiences of criticism and shame had led her to isolate herself. However, facing a collective group response that understood her reaction, neither blamed nor criticized her, but instead provided support and challenged her self-criticism, presented a new type of interaction, diverging from her usual pattern of re-enactment. The process of considering alternative viewpoints prompted Ellen to question her entrenched self-perceptions and assumptions about others, aligning with her treatment goal of adopting a less self-critical approach, and allowed her to question her proneness to withdrawal and explore the possibility to connect with others. At the end of the session, Ellen was invited to formulate for herself a ‘take-home message’ to further reflect upon afterwards, which she formulated as: ‘Try to be more considerate with regard to the responsibility I carry for what has happened in my past. Not everything was my fault, I was still only a child! I can try to stop blaming myself and be more compassionate”.

BOX 7 **Ellen’s second processing session—impact on relationships**

During her second trauma processing session, the group first reflected upon Ellen’s experiences of her first session and the ‘take-home message’ she had generated for herself. She had since used the message repeatedly as reminder to herself of the empathic responses she had experienced and the nuanced perspective this had brought with regards to her felt guilt and responsibility during the first session. She shared that she had been reflecting upon this quite a lot since the first session. Ellen also shared about how listening to the trauma accounts of others since her first session co-facilitated this process of reflection, as hearing others and feeling empathic towards them impacted how she was able to reflect upon her own experiences, further mitigating her feelings of guilt and installing a more empathic understanding towards herself. The session then proceeded to focus on the exploration of the effect of Ellen’s traumatic experiences on her self-perception, her views of others, and the shape of her interpersonal relationships. Ellen discussed her enduring fear of aggression and criticism from others, and how her feelings of unworthiness had hindered her ability to form friendships. She was able to describe her experience of loneliness and sadness due to the absence of positive relationships, and how her experiences of sharing thoughts and feelings within the group helped reinforce her desire for change. Ellen also expressed her perceived obligation to protect her brother, a responsibility she feels even more acutely for her children. Unlike in the past, where such emotions would trigger dissociation (linked to alien self-experiences of shame and self-blame), Ellen now found herself more capable of describing, understanding, and appreciating sharing these feelings with the group. At the end of the session, she added to her previous take-home message: ‘Try to be more considerate with regards to the responsibility I carry for what has happened in my past. Not everything was my fault, I was still only a child! Whenever I start feeling like a failure and disappointment to others, try to take a brief moment to consider whether the other is really being so negatively judging me (and if so, then leave them be and turn to my trusted others!) or whether it is me judging myself and try to be more empathic and less harsh on myself.”

One facilitator guides the patient through exploring the narrative, assisting them in connecting with and discussing the memory. Meanwhile, the co-facilitator focuses on the other group members, ready to intervene and help regulate any distress, whether through eye contact or direct acknowledgment of how expression of the trauma narrative may affect listeners (establishing a we-mode). Throughout this process, both facilitators and group members are committed to adopt a stance of empathetic validation, support, and curiosity, prioritizing the sharing of the narrative over ascribing meaning to the events. While providing reassurance may be instinctive—for instance, in response to expressions of shame (“do not feel like that, you are not like that!”), the facilitators guide the group towards exploring these feelings more deeply rather than foreclosing and invalidating by offering simple reassurances. Group members are discouraged from giving excessive comfort, proposing solutions, or sharing unmentalized perceptions of the perpetrator. Instead, they are encouraged to remain aware of their own reactions while listening to the narrative, while keeping the person that is presenting in mind and considering the impact of the narrated experiences on their perception of the person sharing, particularly in terms of self-view and interpersonal relationships. How does listening change their understanding of the presenting patient? Can they verbalize this change in their perspective on the individual? Can they relate the presenter’s experiences to themselves too (“Now I see them in this way, does that change how I see myself”)? This approach helps reduce the likelihood of psychological avoidance, decreases reliance on distancing behaviors, and enhances engagement with the memory retrieval process.

The act of sharing traumatic experiences can activate intense emotions related to the past, often proving painful and frightening for patients. Narration styles vary, with levels of fragmentation and recall of detail differing significantly among individuals. Memories are frequently fragmented and recalled in states of low mentalizing, heightening the emotional impact and potentially causing considerable distress both for the individual sharing and for other group members. Clinically, managing the arousal levels of both the individual and the group presents a challenge, requiring a variety of approaches such as collaborative arousal regulation through physical exercises, taking brief pauses, providing verbal and non-verbal support, or employing appropriate humour. Therapists balance the optimal level of arousal in order to avoid shifts into prementalizing modes, thereby avoiding the risk of re-traumatization.

Each trauma processing session begins with a review of its structure, including reminders for listeners to seek facilitator assistance as needed and prompts to help the narrator consider necessary support. Therapists also focus on creating a safe and supportive physical environment, such as adjusting seating arrangements for the narrator’s comfort. Patients are urged to think about and communicate how the group might help regulate their distress. Recognizing and explicitly addressing each other’s trauma triggers helps to establish safety time and time again. Interventions aim to foster a sense of agency and empowerment in regulating distress and maintaining safety. Patients might bring personal objects for grounding that help them to remain in their window of tolerance or be reminded of effective self-soothing techniques, like breathing exercises or engaging the senses by focusing on a chosen object.

Therapists remain acutely aware of shifts in the group towards pretend mode. In pretend mode there is a decoupling of mental states from reality and so the discussion lacks focus, and is not rooted within emotion and context. Quick transitioning from working with implicit emotional memory to activating semantic memory (considering general tendency rather than personal experience) may also indicate pretend mode. The sudden shift from expression of feeling to meaning can lead to a decoupling of affect from thought, leading to increasingly general and detached discussions lacking the processing of content with emotional involvement. Therapists strive to minimize the onset of pretend mode, managing participants’ anxiety by leveraging the group’s encouragement, support, and positive reinforcement. While some level of avoidance might be necessary for patients to remain within a mentalizing threshold or ‘window of tolerance’, facilitators ensure avoidance does not become excessive, adjusting the level of exposure to personal experience on the individuals’ ability to connect with their emotions, bodily sensations, and memories, and manage dissociation. Even if patients occasionally exceed their window of tolerance, opportunities for mentalizing about these experiences arise, potentially during a second processing session or as a continued focus throughout treatment.

For those with severe clinical presentations and/or more severe trauma histories, especially individuals who frequently dissociate (including those with but not restricted to dissociative identity disorder), modifications may be necessary, as patients may struggle to access or recount more specific episodes of trauma. Instead of focusing on specific traumatic memories, these patients might be encouraged to discuss events more generally, still aiming to achieve continuity in their personal narratives and social recalibration of their mental processes.

The emphasis on elaborating on thoughts and feelings during the processing sessions encompasses both the domain of understanding the emotions and entrenched beliefs related to the trauma, and, equally crucially, examining how trauma influences interpersonal relationships. Ideally, these areas are explored concurrently, although typically, the focus on mentalizing the trauma narrative precedes a more concentrated examination of its effects on interpersonal dynamics. This approach often results in the initial processing session concentrating more on the trauma narrative itself, while subsequent sessions explore the trauma’s impact on the individual’s (current) interpersonal life, with group members possibly focusing more on one of these aspects at a time. Some patients may choose to introduce a different trauma experience in their second session, possibly influenced by others’ stories or driven by a desire to share something previously withheld. It is important that discussions of the trauma’s impact on relationships and self-other perceptions should not be bypassed. A relational map can be a valuable tool for reflecting on relationships, structured in a manner that minimizes the risk of reactivating traumatic memories. Engaging in mentalizing about how patients perceive themselves and are perceived within the group can prompt insights into potential improvements in relationships outside the treatment context. This reflective process can broaden from a specific focus on trauma to include a wider examination of attachment styles, self and other representations, and behavioral and communicative strategies.

### Phase 3: mourning and loss—generalization and rehabilitation

As the focus shifts from trauma-specific mentalizing to broader considerations of self and relational dynamics, MBT-TF transitions to phase 3. This final phase, which is generally shorter than phase 2, with flexibility depending on the context, begins with a review of the changes that have occurred and the understanding achieved relative to the initial treatment goals and expectations. This review involves both personal reflection and consideration of the progress of other group members. The emphasis then moves to considering the individual’s relationship with the wider world and their present life, aiming to comprehensively shift the focus from past experiences to current reality. The goal is to cultivate a more integrated self-narrative and a coherent self-identity in relation to current and, importantly, also future life, positioning traumatic experiences within the past. This process includes fostering acceptance within self-identity and exploring evolving self-perspectives (see Box 8).

BOX 8 **Moving forward: Ellen’s final phase of acceptance, grief, and further growth**

In the concluding phase, discussions centered on Ellen’s journey through the processing phase, her initial hopes (“relief from all trauma”), the benefits she derived from the group, and aspects that remained unchanged. Ellen experienced grief for her lost childhood and anger towards the events that transpired. Unlike her initial self-directed anger, she now acknowledged her anger as a response to the actions done to her. Additionally, she expressed sadness over her parents’ lack of emotional understanding. Ellen began to feel a sense of compassion towards herself, recognizing the undue responsibility she felt to protect her brother while she was just a child. Gaining a deeper understanding of the pervasive impact of her trauma and her responses allowed Ellen to view her reactions as natural and justified given her experiences. Moreover, Ellen experienced that in everyday life she had on multiple accounts been able to signal feelings of failure and disappointment (i.e., triggered by feedback from colleagues at work), and recognize these feelings as triggers for high arousal, guilt, and shame related to her past experiences. Being able to signal this had led her to feel a bit more in control over these situations, and capable of exploring for herself—and in some instances with trusted others—her experience in these moments, allowing her to connect in a more intimate way with her children and a good friend. She shared her plans to visit her country of birth with her children, aiming to share parts of her story with them, marking a step towards integrating her past with her present and envisioning a future of healing and personal growth.

The conclusion of the group sessions may arouse feelings akin to rejection and can in itself reactivate trauma symptoms or avoidance behaviors. Avoidance is proactively addressed through efforts to ensure commitment, outreach to those who may not attend at this time, and highlighting the significance of a constructive group conclusion. Additionally, the recognition that not all issues have been resolved—and, for some, the desire for more from the group or facilitators—can prompt collective reflections on mourning and loss. The conversation gradually shifts to focus on grief, loss, and mourning over missed life opportunities linked to the trauma. Grief work is multifaceted, involving acceptance of immutable facts (such as lack of support during the traumatic event) and the irrevocable nature of the trauma itself, as well as its lifelong impacts.

Frequently, patients may have denied themselves positive life experiences (e.g., a worthy relationship) due to trauma-induced beliefs about themselves or others. This realization becomes clearer as awareness of trauma re-enactment patterns increases. Phase 3 facilitates navigating these realizations, managing the ensuing distress, and learning to coexist with these realities. Furthermore, discussions about the future encourage patients to contemplate their self-view in managing current and future challenges, set goals for personal development, and decide what learnings from the group they wish to retain in their external relationships. Clinical experience suggests that concluding the group often involves a mix of growth in self-efficacy, appreciation for having shared long-isolated thoughts and emotions, as well as sadness and regret at the group’s end.

## Conclusion

Following the experience of complex trauma, patients frequently encounter profound difficulties related to the enduring effects trauma imposes on mentalizing. The impact on their attachment relationships, emotion regulation, and self-other perceptions combine to impose further limitations of mentalizing, leading to cycles of re-enactment that persistently affect self and interpersonal dynamics. These cycles prevent progress by allowing trauma to dominate present functioning and obstruct future aspirations. MBT-TF introduces a group intervention that mirrors the natural repair and resilience-building mechanisms that can be observed in spontaneous recovery from trauma. It leverages a shared, mentalized viewpoint to mitigate the detrimental impact that trauma has by facilitating the recalibration of trauma-induced experiences, and self and relational beliefs. Whereas a brief series of individual sessions is used to prepare for the trauma-focused work, the group environment is a prerequisite to facilitate the virtuous cycles of recalibrating the traumatized mind. Individual sessions—tailored to the patient and context—may be used as supportive to this key process in group.

To date, two applications of MBT-TF have been introduced in clinical settings: a comprehensive stand-alone MBT-TF program for patients dealing with complex trauma, with or without a concurrent personality disorder diagnosis, and an MBT-TF module designed as an adjunct to existing MBT programs for BPD patients also impacted by complex trauma (Bateman and Fonagy, 2021; Rüfenacht et al., 2023a). Research initiatives, including randomized clinical trials comparing MBT-TF to standard care for individuals with complex PTSD and co-occurring personality disorders, are underway using both these approaches.

Preliminary clinical feedback is encouraging. A recurrent clinical observation has been the significant level of cohesion achieved within groups reported by both clinicians and patients, despite participants presenting with substantial psychopathology and, occasionally, interpersonal tensions arising from trauma re-enactments affecting group dynamics. Yet, engagement and attendance rates have been impressively high. Initial quantitative and qualitative findings from these clinical pilots will be shared in an upcoming publication focused on clinical implementation, highlighting key insights and addressing common challenges, as illustrated through Ellen’s case. Consistent with mentalization-based approaches, peer supervision and consultation have been vital in fostering ongoing reflective practices for clinicians throughout the intervention.

Current pilot studies will inform further refinement and adherence to a definitive MBT-TF manual with better-developed measures of fidelity needed to underpin efficacy trials. Future research should aim to identify specific populations that could benefit most from the trauma-focused interventions described here and investigate patient experiences of therapy and in-session processes through qualitative methodologies to better monitor and understand the underlying mechanisms of change.

## Data availability statement

The original contributions presented in the study are included in the article/supplementary material; further inquiries can be directed to the corresponding author.

## Ethics statement

No potentially identifiable human images or data are presented. To ensure anonymity, the case vignette represents a fictional yet typical patient for the population targeted by the MBT-TF approach.

## Author contributions

MS: Writing – original draft, Writing – review & editing. JV: Writing – original draft, Writing – review & editing. ER: Writing – original draft, Writing – review & editing. LN: Writing – original draft, Writing – review & editing. LS: Writing – original draft, Writing – review & editing. TN: Writing – original draft, Writing – review & editing. PL: Writing – original draft, Writing – review & editing. PF: Conceptualization, Writing – original draft, Writing – review & editing. AB: Conceptualization, Writing – original draft, Writing – review & editing.

## Footnotes

^1^Epistemic trust is a key concept in understanding how people (fail to) learn from and relate to their social environments, through interpersonal communication and relationships. Epistemic trust is developed through consistent, reliable, and positive interpersonal interactions that signal safety, competence, and benevolence, beginning in the context of early childhood within secure attachment relationships, where caregivers respond sensitively and predictably to the child’s needs, continuing into adulthood, as trustworthy interactions encourage individuals to be open to receiving and integrating new information from others. Trauma —and attachment trauma in particular —typically disrupts this process, resulting in persistent epistemic vigilance (mistrust) or epistemic naivety (credulity) that in turn exacerbate negative attachment experiences and hinder the process of resilience in reaction to situations of distress that, in normative development, is provided by social referencing and calibration of one’s mind with the mind of others (social learning). Hence, the cessation of excessive epistemic vigilance and (re-)establishment of epistemic trust are of key importance in psychotherapy in general, and even more so when treating complex trauma, in order to reinstate a process of resilience through social learning (Fonagy et al., 2015, 2019).

^2^Secondary attachment strategies are behaviors and coping mechanisms that individuals develop as adaptive responses to experiences of inconsistency, unavailability, or unresponsiveness from their primary attachment figures. They are categorized into (a) hyperactivating strategies, where individuals seek attention and closeness intensely, often appearing clingy and anxious, seeking constant reassurance and validation; and (b) deactivating strategies, which involve suppressing the need for closeness to avoid emotional pain, leading to emotional distance, self-reliance, and avoidance of intimate relationships (Mikulincer and Shaver, 2012).

^3^Prementalizing modes of experiencing subjectivity reflect ineffective mentalizing that developmentally antedates the capacity for full mentalizing. The mentalizing framework has heuristically identified (1) the psychic equivalence (or ‘thinking inside-out’) mode, characterized by a sameness of what is experienced internally and assumed as external reality and in which thoughts and feelings become too real and the individual can consider no perspectives other than his/her own (‘I think therefore it is fact’; ‘I feel shame, therefore I am shameful’); (2) the teleological (or ‘quick-fix/doing’) mode, characterized by concrete understanding and focus on external reality, in which experience can only be altered by means of concrete actions (‘Actions speak louder than words!’); and (3) the pretend (or ‘bubble’) mode, characterized by a profound sense of disconnection between the acute context/reality and inner experiences that, in the extreme, leads to feelings of derealization and dissociation (Allen and Fonagy, 2010; Luyten et al., 2020a).

^4^‘We-mode’ refers to a state in which two or more individuals achieve mutual understanding of each other’s perspectives, emotions, thoughts, and behaviors as a product of their engaging in joint attention, maintaining their distinct minds while acknowledging their commonalities. It involves seeing the other as a separate yet connected entity, sharing the experience of reciprocity. This shared higher-order mental state is assumed to enhance the ability to understand oneself in a social context and facilitate new ways of understanding and interacting with others (Gallotti and Frith, 2013; Fonagy et al., 2022).

^5^An ‘epistemic match’ pertains to the alignment of compatibility between a patient’s understanding of their own experiences and the model of the mind as provided by the therapist, which allows the patient to feel recognized, understood, and mirrored as an autonomous agentive self by the therapist, fostering epistemic trust and feelings of agency (Fonagy et al., 2019; Fisher et al., 2023).
